# Age trajectories of glycaemic traits in non-diabetic South Asian and white individuals: the Whitehall II cohort study

**DOI:** 10.1007/s00125-014-3448-9

**Published:** 2014-11-28

**Authors:** Satoyo Ikehara, Adam G. Tabák, Tasnime N. Akbaraly, Adam Hulmán, Mika Kivimäki, Nita G. Forouhi, Hiroyasu Iso, Eric J. Brunner

**Affiliations:** 1 Department of Epidemiology and Public Health, University College London, 1-19 Torrington Place, London, WC1E 6BT UK; 2 Public Health, Department of Social and Environmental Medicine, Graduate School of Medicine, Osaka University, Osaka, Japan; 3 First Department of Medicine, Semmelweis University Faculty of Medicine, Budapest, Hungary; 4 Inserm U1061, Université Montpellier II, Montpellier, France; 5 Department of Medical Physics and Medical Informatics, University of Szeged, Szeged, Hungary; 6 MRC Epidemiology Unit, Institute of Metabolic Science, University of Cambridge, Cambridge, UK

**Keywords:** Cohort study, Ethnicity, Fasting glucose, Fasting insulin, Glycaemic trajectory, Insulin resistance, Insulin secretion, Insulin sensitivity, Post-load glucose, South Asian

## Abstract

**Aims/hypothesis:**

South Asian individuals have an increased prevalence of type 2 diabetes, but little is known about the development of glycaemic traits in this ethnic group. We compared age-related changes in glycaemic traits between non-diabetic South Asian and white participants.

**Methods:**

In a prospective British occupational cohort with 5-yearly clinical examinations (*n* = 230/5,749 South Asian/white participants, age 39–79 years at baseline), age-related trajectories of fasting glucose (FG) and 2 h post-load glucose (PLG), log-transformed fasting insulin (FINS) and 2 h post-load insulin (PLINS), HOMA insulin sensitivity (HOMA2-%S) and HOMA insulin secretion (HOMA2-%B) were fitted for South Asian and white individuals who remained free of diabetes between 1991 and 2009.

**Results:**

In sex-adjusted multilevel models, FG was stable in white participants but increased with age in South Asians (0.12 [SE = 0.04] mmol/l per decade). PLG, FINS and PLINS levels were lower among white participants (by 0.271 [SE = 0.092] mmol/l, 0.306 [SE = 0.046] log pmol/l, 0.707 [SE = 0.059] log pmol/l at age 50, respectively) compared with South Asians, although their age-related trajectories were parallel. HOMA2-%S was higher (0.226 [SE = 0.038] at age 50) and HOMA2-%B lower (by 0.189 [SE = 0.026] at age 50) among white than South Asian participants. The age-related decline in HOMA2-%S was similar in these groups, but the age-related increase in HOMA2-%B was greater in white participants (0.04 [SE = 0.02] per decade). This difference was explained by obesity, lifestyle and social status.

**Conclusions/interpretation:**

Findings from a diabetes-free population suggest an inadequate pancreatic beta cell reserve in South Asians, as a significantly steeper age-related increase in FG was observed in this ethnic group compared with white individuals.

## Introduction

The prevalence of diabetes is high and has dramatically increased among native and migrant Asian populations [1, 2]. Approximately 20% of all individuals with diabetes (~71 million people) live in South Asia (Bangladesh, India, Nepal, Pakistan and Sri Lanka), with the prevalence of diabetes estimated to increase over the next decade [3]. Studies show that South Asians have a twofold to fivefold elevated risk of type 2 diabetes compared with white individuals in the UK [4–6].

Several potential explanations for an elevated risk of diabetes among South Asians compared with white individuals have been proposed [1, 2, 7, 8]. The case for reduced insulin sensitivity is well established in South Asians from childhood to adulthood, based on measures such as fasting insulin (FINS) or HOMA insulin sensitivity (HOMA2-%S), which are directly comparable across populations [9–16]. The role of insulin secretion remains equivocal [12, 14]. Reduced insulin secretion (or pancreatic functional reserve) is a plausible aetiological factor in South Asians because fetal undernutrition is common in Asian populations and may lead to reduced growth of the pancreas [1]. However, beta cell function is difficult to study because measures of insulin secretion such as HOMA insulin secretion (HOMA2-%B) and the insulinogenic index do not account for the dependence of insulin secretion on blood glucose level and the underlying insulin sensitivity [17, 18]. This complex association could be characterised by the disposition index, although its estimation is invasive and labour intensive, and it is therefore rarely used in population-based studies [17, 18].

One way to overcome the barrier of estimating beta cell function is to describe parallel changes in insulin sensitivity and insulin secretion over time. We previously identified compensatory changes in insulin secretion among adults before diabetes diagnosis using this approach [19]. We hypothesised that, given an age-related decline in insulin sensitivity [19], the compensatory increase in insulin secretion would be less in South Asians than in white individuals, leading also to an increase in fasting glucose (FG), which supports the hypothesis of a reduced pancreatic functional reserve in South Asians. To study these questions, we assessed (1) age-related trajectories of FG, 2 h post-load glucose (PLG), FINS, 2 h post-load insulin (PLINS), insulin sensitivity and insulin secretion among non-diabetic South Asian and white participants using longitudinal data from the Whitehall II study, and (2) investigated potential mechanisms that might explain differences in these trajectories between South Asian and white populations.

## Methods

### Study population and design

The Whitehall II study was initiated in 1985 and recruited 10,308 participants (3,413 women) aged 35–55 years, with a response rate of 73%, from 20 London-based Civil Service departments [20]. The initial visit (phase 1) included a clinical examination and a self-administered questionnaire in 1985–1988. During the follow-up, 5-yearly clinical examinations were performed (Phase 3: 1991–1994; Phase 5: 1997–1999; Phase 7: 2002–2004; Phase 9: 2007–2009) and additional postal questionnaire-only phases were conducted (Phase 2: 1988–1990; Phase 4: 1995–1996; Phase 6: 2001; Phase 8: 2006). The University College London Medical School Committee on the Ethics of Human Research provided ethical approval for the study, and written informed consent was obtained from all participants.

**Fig. 1:**
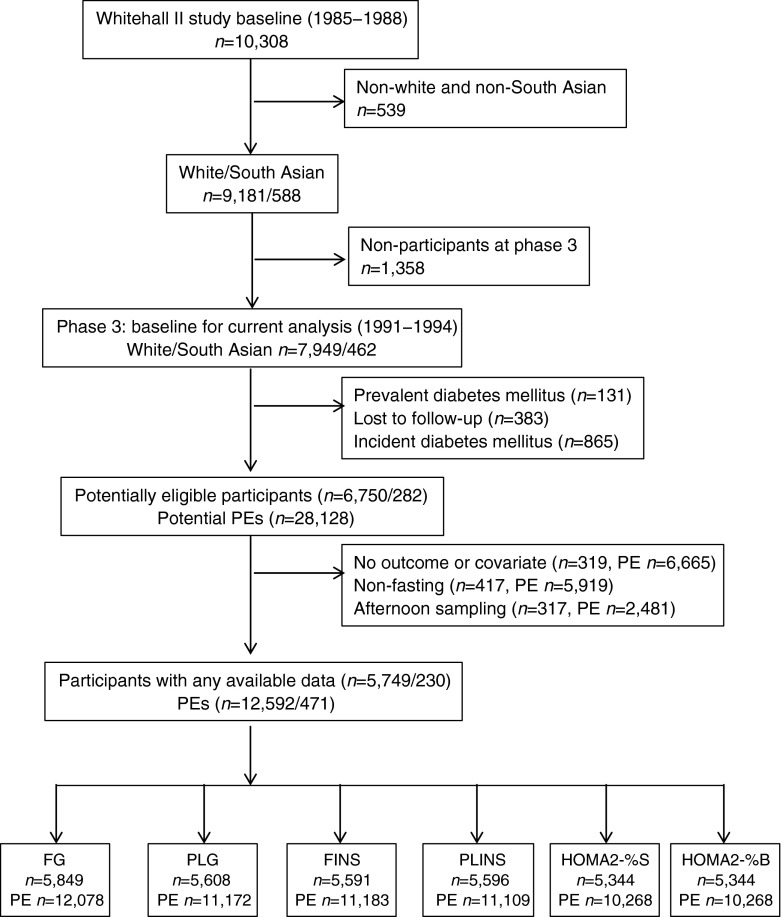
Flow chart of the selection of participants for the current study. PE, person-examination

As a 75 g OGTT was first performed in phase 3; this provides the baseline for the current analysis. Of the 8,411 South Asian and white participants at baseline, 7,032 (282 South Asian and 6,750 white participants) remained free of diabetes during the follow-up and were eligible for the present study. After the exclusion of 319 participants with unavailable glucose levels, insulin values or missing covariates, 417 non-fasting (<8 h) participants, and 317 persons because of an afternoon sampling, the final dataset included 5,979 participants (230 South Asian and 5,749 white, i.e. 13,063 person-examinations) (Fig. 1).

Of the potential 28,128 person-examinations that included four repeat measures for each of the 7,032 eligible participants, we excluded 6,665 observations with unavailable glucose and insulin values or missing covariates, 5,919 observations with non-fasting sampling (<8 h) and 2,481 observations due to afternoon sampling. Thus, the final dataset included 13,063 person-examinations for the 5,979 participants who took part in at least once of the four examinations every 5 years. Data were used for 87 South Asian participants with one observation, 70 with two observations, 48 with three observations and 25 with four observations, and for 1,692 white participants with one observation, 1,898 with two observations, 1,532 with three observations and 627 with four observations (Fig. 1).

### Measurements

Fasting and 2 h post-load venous blood samples were taken during a 75 g OGTT according to standardised protocols during all phases of the study. Blood glucose was measured with the glucose oxidase method (YSI Corporation, Yellow Springs, OH, USA). Serum insulin was measured with an in-house human insulin RIA and later with a DAKO ELISA kit (Dako Cytomation Ltd, Ely, UK) [19].

Prevalent cases of diabetes (South Asian *n* = 31, white *n* = 100) at phase 3 as well as incident cases were excluded based on a diagnosis either by an FG ≥7.0 mmol/l or a PLG ≥11.1 mmol/l during the OGTT (South Asian *n* = 40, white *n* = 391), by the use of glucose-lowering medication (South Asian *n* = 21, white *n* = 108) or by reporting doctor-diagnosed diabetes (South Asian *n* = 40, white *n* = 263) at screening or during the additional questionnaire phases [19].

HOMA2-%S and HOMA beta cell function (HOMA2-%B, a marker of insulin secretion) were calculated with the HOMA2 calculator version 2.2 (www.dtu.ox.ac.uk/homacalculator/index.php) using FG (acceptable range 3–25 mmol/l) and FINS (acceptable range 20–400 pmol/l) measurements [21]. The BMI (the body weight in kilograms divided by the height in metres squared [kg/m^2^]) was measured according to standardised protocols.

Ethnicity was defined according to the Office for National Statistics 1991 census types. Self-reported ethnicity at phase 5 was mainly used; missing data were complemented by observer-assigned ethnicity from phase 1 [22]. The rates of agreement between observer-assigned and self-reported ethnicity were high (93% for South Asian and 99.3% for white individuals). South Asians were defined as participants of Indian, Sri Lankan, Pakistani or Bangladeshi ethnic origin.

British Civil Service employment grade was used as a measure of occupational position and was grouped into three categories: high (senior administrators), intermediate (executives, professionals and technical staff) and low (clerical and office support staff) [20].

Physical activity was assessed by the answers to questions on the frequency and duration of participation in moderate or vigorous physical activity at phase 3. At phases 5, 7 and 9, these questionnaires included 20 items on the frequency and duration of different physical activities that were used to calculate the hours per week spent at each intensity level. Physical activity levels were classified as active (≥2.5 h/week of moderate or ≥1 h/week of vigorous physical activity), inactive (≤1 h/week of moderate and ≤1 h/week of vigorous physical activity) or moderately active (if not active or inactive) [20].

Dietary patterns were based on the type of bread and milk most frequently consumed and the frequency of fruit and vegetable consumption (on an 8-point scale). First, each indicator was scored from 1 to 3 points. For the type of bread, this was: healthy (wholemeal, wheat meal or other brown bread) = 1, moderately healthy (both healthy and unhealthy) = 2 or unhealthy (white bread) = 3. For the type of milk, the scoring was: healthy (no milk, skimmed milk or other) = 1, moderately healthy (semi-skimmed milk) = 2 or unhealthy (whole milk) = 3. For the frequency of fruit and vegetable consumption, the categories were: healthy (daily or ≥2 times per day) = 1, moderately healthy (≥1 times per week) = 2 or unhealthy (<3 times per month) = 3. The dietary score was calculated as the sum of the previous points classified into three groups: healthy (3 points), moderately healthy (4–7 points) or unhealthy (≥8 points) [23].

### Statistical analysis

Two-sample *t* tests and *χ* ^2^ tests were conducted to compare means and proportions, respectively, at baseline between South Asian and white individuals. FINS and PLINS levels, HOMA2-%S and HOMA2-%B were natural log-transformed because of their skewed distribution.

Linear mixed models were used to assess age-related trajectories of glycaemic traits including FG, PLG, FINS, PLINS, HOMA2-%S and HOMA2-%B. These models use all available data and take into account the interrelationship between within-individual data points. First, we modelled differences in trajectories by ethnicity (South Asian or white) using the participants’ age (centred at 50 years) as the underlying time variable adjusted for sex, including ethnicity, age, age squared and their interaction terms and sex into the model. As the age squared by ethnicity interaction did not improve the model fit and the term itself was not significant, we dropped this term from all the models. Furthermore, our results suggested that the FG trajectory was following a linear increase, and thus the quadratic age term was also dropped from this model.

Second, we further adjusted for occupational grade and time-varying BMI, physical activity and dietary score. For easier comparability of the models, we used the same specification for these multivariable adjusted models as for the sex-adjusted model.

Due to the fact that some people had available data on only one of the six glycaemic traits used for analysis, the final number of participants for the individual glycaemic traits is lower than of the total population (*n* = 5,979): 5,849 for FG, 5,608 for PLG, 5,591 for FINS, 5,596 for PLINS and 5,344 for HOMA2-%S and HOMA2-%B (Fig. 1).

All statistical analyses were performed in Stata 12.1 (StataCorp, College Station, TX, USA), and statistical significance was inferred at a two-tailed *p* < 0.05.

## Results

Table 1 shows the baseline characteristics by ethnicity of the participants who remained free of diabetes during the follow-up. Compared with their white counterparts, South Asian participants were 1.5 years older and more likely to be female. They had 0.33 mmol/l higher PLG, 32.5% higher FINS, 110.7% higher PLINS, 18.3% lower HOMA2-%S and 18.3% higher HOMA2-%B levels. They were more likely to have low physical activity, follow an unhealthy diet and have a low occupational grade. There was no significant difference in BMI and FG between South Asians and white individuals.

**Table 1:** Baseline characteristics of study participants by ethnicity at the baseline examination

Variable	White (SD)	South Asian (SD)	*p* values
*n*	5,749	230	
Age (years)	52.4 (7.7)	53.9 (7.4)	0.003
Male	71.5%	62.6%	0.003
BMI (kg/m^2^)	25.4 (3.7)	25.1 (3.6)	0.08
FG (mmol/l)	5.20 (0.47)	5.17 (0.50)	0.11
PLG (mmol/l)	5.22 (1.36)	5.55 (1.32)	0.0001
FINS (pmol/l)^a^	41.6 (12.2–141.9)	55.2 (17.2–176.6)	<0.0001
PLINS (pmol/l)^a^	196.2 (39.5–974.1)	413.3 (102.5–1667.6)	<0.0001
HOMA2-%S^a^	113.2 (42.0–305.4)	92.5 (32.0–267.1)	<0.0001
HOMA2-%B^a^	80.2 (41.0–157.1)	95.0 (45.5–197.9)	<0.0001
Low physical activity	21.8%	42.2%	<0.0001
Unhealthy dietary pattern	4.8%	10.1%	<0.0001
Low occupational grade	11.4%	35.2%	<0.0001

Data are mean (SD), geometric mean (95% CI) or percentages

Comparisons were made using two-sample *t* tests or *χ* ^2^ tests, as appropriate

HOMA2-%S and HOMA2-%B were calculated using HOMA2 calculator version 2.2 (Diabetes Trials Unit, University of Oxford, Oxford, UK) [23]

^a^Log-transformed before analysis due to the skewed distribution

**Fig. 2:**
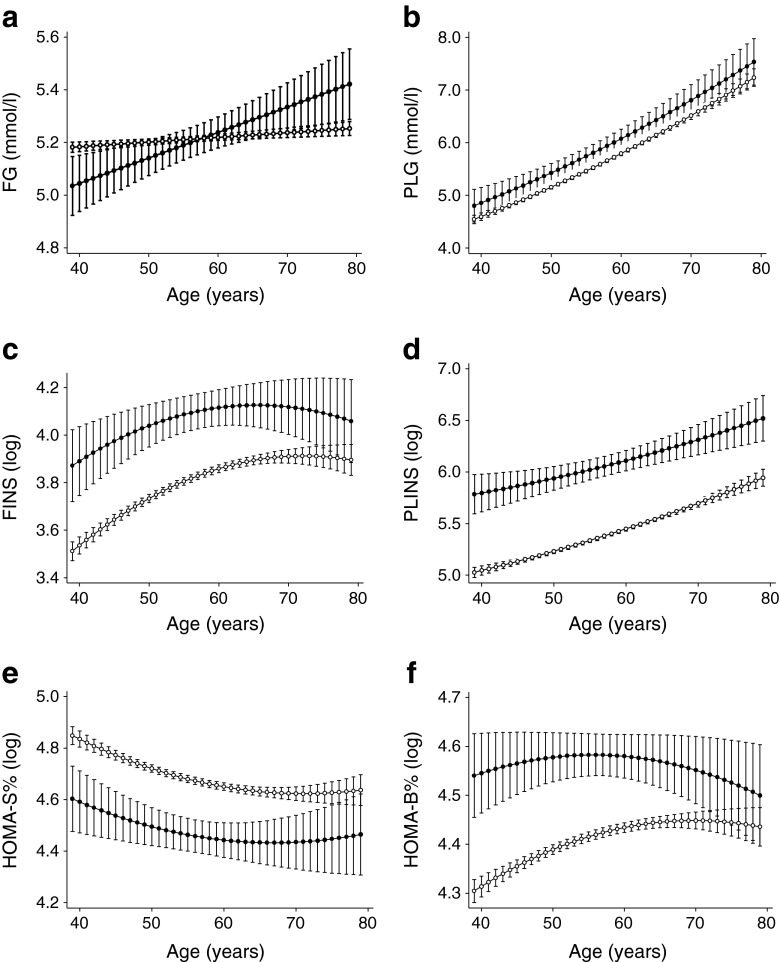
Estimated trajectories of glycaemic traits by age and ethnicity adjusted for sex: (a) FG, (b) PLG, (c) FINS, (d) PLINS, (e) HOMA2-%S and (f) HOMA2-%B. Error bars show 95% CI around fixed effects. Black circles, South Asian participants; white circles, white participants. HOMA2-%S and HOMA2-%B were calculated using HOMA2 calculator version 2.2 (Diabetes Trials Unit, University of Oxford, Oxford, UK) [23]

Figure 2a shows the age-related trajectories of FG by ethnicity adjusted for sex. FG followed a steep, linearly increasing age trajectory among South Asians (0.12 [SE = 0.04] mmol/l per decade). In contrast, the trajectory of FG was less steep (by 0.11 [SE = 0.04] mmol/l per decade, *p*_interaction_ < 0.01) for white individuals. Further adjustment for diet, physical activity and job grade had no major effect on the difference in the slope (Table 2).

**Table 2:** Fixed effects for linear mixed models of change for FG, PLG, FINS, PLINS, HOMA2-%S and HOMA2-%B as outcomes with age as the underlying time variable

Variable	FG (mmol/l)	PLG (mmol/l)	FINS (log)	PLINS (log)	HOMA2-%S (log)	HOMA2-%B (log)
Participants (*n*)	5,849	5,608	5,591	5,596	5,344	5,344
Person-examinations (*n*)	12,078	11,172	11,183	11,109	10,268	10,268
Sex-adjusted						
White	0.085 (0.044)	−0.271 (0.092)**	−0.306 (0.046)***	−0.707 (0.059)***	0.226 (0.038)***	−0.189 (0.026)***
Age	0.012 (0.0035)***	0.067 (0.0086)***	0.011 (0.004)	0.019 (0.005)***	−0.007 (0.003)*	0.002 (0.002)
White × age	−0.011 (0.0035)**	−0.001 (0.008)	0.005 (0.0036)	0.005 (0.0045)	−0.002 (0.003)	0.004 (0.002)*
Age^2^	–	0.0004 (0.0001)**	−0.0004 (0.00006)***	0.0002 (0.00008)***	0.0002 (0.00006)***	−0.0002 (0.00004)***
Multivariable adjusted						
White	0.173 (0.076)*	−0.256 (0.092)**	−0.308 (0.04)***	−0.669 (0.058)***	0.233 (0.033)***	−0.236 (0.030)***
Age	0.0087 (0.0027)*	0.062 (0.0085)***	0.004 (0.003)***	0.016 (0.005)***	−0.003 (0.003)	−0.0011 (0.0020)
White × age	−0.009 (0.0027)**	−0.004 (0.008)	0.0009 (0.003)	0.002 (0.004)	0.002 (0.003)	0.002 (0.002)
Age^2^	–	0.0006 (0.0001)***	−0.00002 (0.00005)	0.0004 (0.00007)***	−0.00005 (0.00005)	0.00001 (0.00003)

Multivariable adjusted: adjustment for BMI, sex, dietary score, physical activity and occupational grade

Age centred at 50 years

HOMA2-%S and HOMA2-%B were calculated using the HOMA2 calculator version 2.2 (Diabetes Trials Unit, University of Oxford, Oxford, UK) [23]

*p < 0.05, **p < 0.01, ***p < 0.001

While the PLG was lower (by 0.271 [SE = 0.092] mmol/l at age 50) among white compared with South Asian participants throughout the follow-up, the parabolic shape of the age-related trajectories was similar between ethnicities (absolute slope difference 0.01 [SE = 0.08] mmol/l per decade) (Fig. 2b). Further multivariable adjustment had no major effect on these terms (Table 2).

White participants had lower FINS and PLINS values (by 0.306 [SE = 0.046] and 0.707 [SE = 0.059] log pmol/l at age 50) across the age range compared with South Asians. Sex-adjusted age-related trajectories of FINS and PLINS followed a quadratic trend that became non-significant for FINS in the multivariate model (Fig. 2c, d and Table 2).

HOMA2-%S was higher (0.226 [SE = 0.038] at age 50) and HOMA2-%B lower (by 0.189 [SE = 0.026] at age 50) among white participants throughout the follow-up. Both HOMA values followed a quadratic age trajectory. HOMA2-%S declined similarly in white and South Asian individuals (absolute slope difference 0.02 [SE = 0.03] per decade). HOMA2-%B increased more quickly (0.04 [SE = 0.02] per decade) in white individuals in response to the decline in insulin sensitivity compared with South Asians in the sex-adjusted model. After multivariable adjustment, the quadratic trend became non-significant in both HOMA measures and showed parallel decreases for HOMA2-%S. The slope difference of HOMA2-%B decreased to non-significance in the multivariable model; however, the actual slope term was negative (decreasing) for South Asians and positive (increasing) for white participants (Fig. 2e, f and Table 2).

## Discussion

The analysis of age trajectories for glycaemic traits suggests inadequate insulin secretion among South Asians, leading to rising FG levels. In white individuals, insulin secretion showed compensatory increases in line with decreasing insulin sensitivity, providing stable FG values. These findings support the hypothesis that although South Asians have higher compensatory insulin secretion at younger ages, they are unable to produce further beta cell compensation in response to decreasing insulin sensitivity above 60 years of age. The major strength of this study is the use of the repeat measures of glycaemic traits to compare age-related trajectories among South Asian and white individuals over a wide age range.

We found that FG remained stable in white participants, whereas it increased rapidly with age in South Asians. PLG was higher among South Asians compared with white participants, but the respective quadratic trajectories were similar. Furthermore, we observed that South Asians had higher FINS and PLINS levels, with parallel increases with ageing in both ethnicities. HOMA2-%S was lower among South Asian compared with white individuals and decreased similarly in both groups. HOMA2-%B was increased in South Asians at baseline, but they responded with a reduced compensatory increase despite similar decreases in HOMA2-%S compared with whites. These data suggest that long-standing decreased insulin sensitivity coupled with impaired beta cell compensation provides the pathophysiological basis of the higher susceptibility to an earlier onset of type 2 diabetes in this cohort of South Asian compared with white individuals.

The finding of constant fasting blood glucose with ageing in whites confirms our previous observation in the same cohort using fewer time points and a shorter follow-up [19]. The population-based cross-sectional Diabetes Epidemiology: Collaborative analysis Of Diagnostic criteria in Asia (DECODA) study reported increases of 0.09–0.13 mmol/l per decade of age for FG in different Asian populations, which corresponds well to the slope of 0.11 mmol/l per decade observed in the current study [12]. As we excluded participants with incident diabetes during the follow-up, the participants included represent a healthy selection of the Whitehall II population such that (1) the true population trajectories are likely to be steeper and (2) the observed lower FG levels among South Asians at study baseline may reflect the selection criteria.

PLG followed a quadratic increase over time, highlighting that additional data waves improved the precision of the estimation over our previous analysis, where a linear age-related increase was observed (0.5 mmol/l per decade) [19]. Other studies found increased PLG levels among non-diabetic South Asian compared with white individuals [10, 14, 16]. We confirm and extend this observation by showing that this difference is constant over the age range of 40–80 years.

FINS and PLINS levels were both elevated in South Asians compared with white individuals, reflecting increased insulin resistance among the South Asian participants [10, 11, 14, 16]. As with PLG, we confirm these findings and extend them by reporting parallel insulin trajectories in South Asians and white participants.

The trajectories of HOMA2-%S, a measure of muscle and hepatic insulin sensitivity, confirm that insulin sensitivity decreases with age. This decrease is less steep at older ages than in middle-aged people, as reflected by the quadratic term in the model. South Asian participants had lower insulin sensitivity over all ages, confirming findings from the literature that shows decreased insulin sensitivity from a young age in South Asians, decades before the manifestation of diabetes [10, 11, 14–16]. This fact may be one explanation for our finding that current lifestyle explained very little of the difference in FG and HOMA2-%S.

The plausible causes for the early emergence of decreased insulin sensitivity among South Asians include the contribution of abdominal fat distribution and low muscle mass [1, 2, 4, 7–9, 13, 14]. Furthermore, levels of the insulin-sensitising hormone adiponectin are lower [24], while levels of proinflammatory cytokines are higher in South Asian compared with white individuals [25, 26]. In addition, unhealthy lifestyles (low physical activity and a Western dietary pattern) could further decrease insulin sensitivity in South Asian children and adults [1, 2, 4, 5, 7, 8, 27]. In our multivariable analysis, we adjusted for obesity (BMI), diet and physical activity, but the main differences between the South Asian and white participants remained, in terms of both the slope difference in FG and the large intercepts regarding PLG, insulin and insulin sensitivity. These findings were robust to adjustment for waist circumference (a measure of central obesity) rather than BMI in a sensitivity analysis.

In line with other investigations, we found increased HOMA2-%S among South Asian compared with white individuals [14]. However, elevated insulin secretion is difficult to interpret since (unlike the disposition index) it does not take account of the underlying level of insulin resistance, and is thus unable to provide a precise indicator of beta cell function. None of the previous studies reported repeat measures of insulin secretion together with glucose values and levels of insulin sensitivity in South Asian and white individuals and was thus unable to compare beta cell compensation across ethnicities. Our results, however, clearly show that, for similar decreases in insulin sensitivity over time in the South Asian and white populations, the white participants showed a larger insulin secretory response and could maintain stable FG levels, while the South Asians could not increase their insulin secretion adequately, explaining the increasing FG trajectory. It is possible that (1) significant fetal undernutrition among Asian populations may lead to reduced beta cell reserve, limiting the compensatory capacity [1], (2) long-term beta cell compensation for chronic insulin resistance from childhood leads to beta cell exhaustion or (3) increasing FG levels and continuously increased post-load glucose produce glucose toxicity and consequently beta cell failure [17, 18]. Although all three of the above mechanisms are compatible with our findings, it should be noted that our data are only hypothesis-generating and are unable to prove or disprove these mechanisms. Although it is well accepted that progression to type 2 diabetes is associated with impaired beta cell compensation some 3–4 years before the diagnosis of diabetes [17, 18], it is remarkable that impaired beta cell function was found in South Asian people who remained diabetes-free throughout the whole follow-up period of the Whitehall II study.

After multiple adjustments for obesity, lifestyle factors and social status, the ethnic difference in beta cell compensation over time was attenuated, suggesting that lifestyle interventions could help to prevent these deleterious changes. Although insulin secretion appeared to develop in parallel in South Asian and white individuals, the difference in slope for FG still remained, confirming that lifestyle factors might not fully explain the deteriorating glucose tolerance among South Asians. While lifestyle interventions are proven in diabetes prevention among prediabetic individuals (individuals with intermediate hyperglycaemia) [18], our study suggests a potential role for them even in healthy South Asians.

The present study has some limitations. First, we used BMI rather than waist circumference as a measure of adiposity because BMI is highly reproducible and has a similar distribution in each sex. Furthermore, the correlation between BMI and waist measurement was strong in our database, with *r* values of 0.73–0.82 in subsequent clinical examinations, and BMI predicted the risk of diabetes as well as waist circumference in a meta-analysis [28]. Second, we used self-reported physical activity and dietary habits instead of objective measures (accelerometry and nutritional biomarkers) as covariates in our analysis, as the variables used in the present analysis have already been shown to be associated with risk of type 2 diabetes [20] and all-cause mortality [23] in the Whitehall II study. Third, as our analysis is based on an occupational cohort and does not include blue-collar workers and the unemployed, the findings may not be fully applicable to the general population. Furthermore, the low number of South Asian participants further limits the generalisability. It should be noted, however, that previous reports from the same study confirmed the role of known diabetes risk factors (including ethnicity) with similar associated relative risks as have been found in population-based studies, giving credibility to the present observations [20, 29–31]. Finally, several other potential confounding factors such as birthweight, birth place for South Asians (Asia or UK), inflammatory markers, adipokines and psychological factors were not taken into account in the present analysis.

In conclusion, the present study is the first to describe ethnic differences in age-related changes of glycaemic traits that may provide plausible pathophysiological mechanisms for the elevated risk of diabetes among South Asians. Specifically, non-diabetic South Asian individuals had lower insulin sensitivity from middle age onwards and inadequate beta cell compensation at older ages compared with white participants. Our results may partially explain the dramatically increased risk of type 2 diabetes among South Asians, and they also highlight the importance of primary prevention of diabetes through lifestyle intervention among South Asian populations.

## Abbreviations

FG: Fasting glucose
FINS: Fasting insulin
HOMA2-%S: HOMA of insulin sensitivity
HOMA2-%B: HOMA of insulin secretion
PLG: 2 h post-load glucose
PLINS: 2 h post-load insulin
